# Cytotoxicity of PLGA-zinc oxide nanocomposite on human gingival fibroblasts

**DOI:** 10.34172/japid.2023.010

**Published:** 2023-06-03

**Authors:** Asieh Mozaffari, Samira Mohammad Mirzapour, Motahare Sharifi Rad, Mehdi Ranjbaran

**Affiliations:** ^1^Department of Periodontics, Faculty of Dentistry, Qazvin University of Medical Sciences, Qazvin, Iran; ^2^Department of Periodontics, Faculty of Dentistry, Tabriz University of Medical Sciences, Tabriz, Iran; ^3^Department of Pharmaceutical Nanotechnology, School of Pharmacy, Zanjan University of Medical Sciences, Zanjan, Iran; ^4^Metabolic Diseases Research Center, Research Institute for Prevention of Non-Communicable Diseases, Qazvin University of Medical Sciences, Qazvin, Iran

**Keywords:** Cell survival, Fibroblasts, Nanoparticles, Polylactic acid‒polyglycolic acid copolymer, Zinc oxide

## Abstract

**Background.:**

Polylactic-co-glycolic acid and zinc oxide (PLGA-ZnO) nanocomposite has been investigated for its antibacterial properties, which could be beneficial for adding to wound dressings after periodontal surgery. However, its cytotoxicity against human gingival fibroblasts (HGFs) remains unclear and should be evaluated.

**Methods.:**

ZnO nanoparticles were synthesized using the hydrothermal method. These metallic nanoparticles were incorporated into the PLGA matrix by the solvent/non-solvent process. The nanomaterial was evaluated by field emission scanning electron microscopy (FESEM), Fourier transform infrared (FTIR), thermogravimetric analysis (TGA), and x-ray diffraction (XRD) analyses. HGF cells were acquired from the National Cell Bank and categorized into four groups: ZnO, PLGA, ZnO-PLGA, and control. The cells were exposed to different ZnO (1, 20, 40, 60, 80, and 100 µg/mL) and PLGA (0.2, 4, 8, 12, 16, and 20 µg/mL) concentrations for 24 and 48 hours. The cytotoxicity was tested using the MTT assay. The data were analyzed using SPSS 25, and *P*<0.05 was considered statistically significant.

**Results.:**

ZnO nanoparticles exhibited significant toxicity at≥40 µg/mL concentrations after 24 hours. Cell viability decreased significantly at all the tested concentrations after 48 hours of exposure. PLGA-ZnO cell viability in 24 hours was similar to the control group for all the concentrations up to 80 µg/mL.

**Conclusion.:**

ZnO nanoparticles could be toxic against HGF in high concentrations and with prolonged exposure. Therefore, incorporating ZnO nanoparticles into a biocompatible polymer such as PLGA could be a beneficial strategy for reducing their toxicity.

## Introduction

 Proper wound healing is essential in the oral cavity because it seals the underlying tissues against pathogenic bacteria and leads to the reconstruction of damaged tissues. Gingival fibroblasts play a fundamental role in healing by creating a new collagen-rich matrix.^1-3^ Because of their antibacterial qualities, various metallic nanoparticles such as gold, silver, titanium, and zinc oxide (ZnO) have been proposed to be incorporated into wound dressings to facilitate healing.^2,4-6^ The surface-to-volume ratio of ZnO nanoparticles is more significant than their bulk form, resulting in enhanced surface reactivity and improved dispersion. This has the potential to be a double-edged sword. Because of their nanometric scale, nanoparticles can easily infiltrate the cell wall. Although a higher surface-to-volume ratio can result in more effective antibacterial characteristics, it can also lead to significant cytotoxicity against normal cells such as fibroblasts. Due to their high intrinsic toxicity, ensuring the safety of these nanoparticles against fibroblasts is crucial so that they do not interfere with the normal healing process.^7-9^

 Two critical factors influencing the cytotoxicity of ZnO nanoparticles are the concentrations used and the duration of cell exposure. When the duration of exposure is increased from 6 to 48 hours, and the concentrations employed are > 50 µg/mL, the cytotoxicity of these nanoparticles against periodontal ligament (PDL) and dermal fibroblasts increases.^10^

 Various natural or synthetic polymers have been discussed in the literature to produce biocompatible and biodegradable nanoparticles. Natural polymers such as chitosan or silk fibroin can be used as a metallic nanoparticle carriers. The primary disadvantage while using natural polymers is the lack of batch-to-batch consistency, which hinders reproducibility and flexibility in the manufacturing process.^8,11,12^ However, we can overcome this issue by using synthetic polymers. Polylactic-co-glycolic acid (PLGA) is a synthetic biodegradable copolymer approved by the Food and Drug Administration (FDA) for drug delivery because of its controlled biodegradability. Its biodegradation rate can be manipulated by altering the percentage of lactic acid. If the copolymer contains more lactic acid, its degradation process will be prolonged. PLGA with 50:50 composition is composed of equal proportions of lactic and glycolic acids. PLGA 50:50 is the most popular composition in nanomedicine due to its rapid biodegradation rate of approximately 50‒60 days.^9,13-16^

 ZnO nanoparticles have been advocated for use in wound dressings because of their antibacterial properties. However, given their high intrinsic toxicity, ensuring the safety of these nanoparticles against HGF cells is essential. Therefore, incorporating ZnO nanoparticles into a biocompatible polymer such as PLGA might be a beneficial strategy for reducing the toxicity of these metallic nanoparticles. This study aimed to test this hypothesis by assessing the cytotoxicity of PLGA-ZnO nanocomposite against human gingival fibroblasts (HGF) with 3-(4,5-Dimethylthiazol-2-yl)-2,5-diphenyltetrazolium bromide (MTT) assay.

## Methods

###  Materials 

 PLGA 50:50 Resomer RG504H with a molecular weight of 48 kDa was purchased from Sigma- Aldrich Company (Sigma-Aldrich, St. Louis, USA). RPMI-1640 medium and 10% fetal bovine serum (FBS) were obtained from the Gibco Company (Gibco, USA). In addition, human gingival fibroblasts (C10459) were acquired from the National Cell Bank (Pasteur Institute, Iran).

###  Synthesis of ZnO nanoparticles

 ZnO nanoparticles were prepared by the hydrothermal method.^17,18^ The process was started by adding an 0.1-mol solution of sodium hydroxide (NaOH) to an 0.05-mol solution of zinc acetate dihydrate (Zn (CH_3_COO)_2_.2H_2_O) on a magnetic stirrer (IKA, Germany). The prepared suspension was placed in the autoclave (Memmert, Germany) at 160°C for 8 hours. The final product was cooled until it reached room temperature. Subsequently, it was centrifuged using Optima XPN-100 ultracentrifuge (Beckman Coulter, USA) at 3000 rpm. The synthesized white residue was washed out with ethanol and deionized water several times and dried at 90°C in the oven (Memmert, Germany).

###  Synthesis of PLGA- ZnO nanocomposite

 The metallic nanoparticles were incorporated into the polymeric matrix by dissolving 200 mg of PLGA in 10 mL of acetone as the solvent while being mixed constantly on the stirrer for 15 minutes at room temperature. Then, 1 mL of ZnO in acetone (0.55% w/v) was added to the PLGA-acetone solution while stirring for 30 minutes at 1200 rpm. After that, 15 mL of ethanol was added as the non-solvent to precipitate the solution, which was then gradually poured into 40 mL of 0.05% w/v polyvinylpyrrolidone (PVP) solution while being constantly mixed at 1200 rpm. PVP was employed as a stabilizing agent to reduce the aggregation of the particles. Finally, the decanted PLGA-ZnO dispersion was allowed to dry at room temperature overnight.^18^

###  FESEM analysis

 The samples were treated in an ultrasonic bath for 15 minutes. Then the dimensions and morphology of the synthesized nanoparticles were evaluated by field emission scanning electron microscopy performed with FESEM Sigma 500 VP (ZEISS Sigma, USA) with a resolution of 1.3 nm at 1 kV.

###  XRD analysis

 To identify the phase composition of the samples and determine their crystalline or amorphous structure, x-ray diffraction was utilized with XRD PW1730/10 (Philips, Netherlands) in the 2θ range of 0‒80° with 0.05° scanning step width performed 2 seconds for each step.

###  FTIR analysis

 FTIR spectroscopy was carried out by Tensor 27 (Bruker, Germany) using wave numbers in the range of 0‒4000 cm^-1^ to confirm the chemical composition of the synthesized nanomaterials and identify their spectral properties.

###  TGA analysis

 TGA was performed with Q600 SDT (TA Instruments, USA) on samples of 15 mg. This analysis was performed to assess the alterations in the stability of the samples at different temperatures. The temperature range was 0‒800 °C. The data were adjusted in the baseline by executing a blank run that would be subtracted from the original data.

###  Treatment of cells with nanoparticles

 RPMI 1640 medium containing 10% fetal bovine serum, 100 000 IU/L of penicillin, and 0.1 g/L of streptomycin was used for the cell cultures. They were placed in 96-well plates inside the incubator (Memmert, Germany) at 37 °C with 5% CO_2_ for 24 hours to attach. Then the medium was discarded, and the cells were divided into 3 test groups and one control group. The first test group was cultured with different concentrations of ZnO nanoparticles (1, 20, 40, 60, 80, and 100 µg/mL). The second test group was exposed to PLGA (0.2, 4, 8, 12, 16, and 20 µg/mL), and the third test group was cultured with PLGA-ZnO nanocomposite with the concentrations mentioned above for 24 and 48 hours. In the control group, the cells were cultured without any nanoparticles.

###  Cytotoxicity evaluation

 The cytotoxicity of the samples was quantified using the MTT assay. Viable fibroblasts can reduce the yellow tetrazolium dye to insoluble formazan with a purple color. The intensity of this color reflects the number of vital cells. After treating HGF cells with different concentrations of nanoparticles, the medium in the wells was removed, and 0.05 mg/0.1 mL of MTT was added to each well. Then the cells were incubated for 3 hours. Subsequently, the formazan salts were dissolved using 0.04 mol of hydrochloric acid in isopropanol, 0.1 mL in each well. Light absorption was quantified by an ELISA Reader (BD Biosciences, USA) at 570 nm. The data were analyzed with SPSS 25 (IBM, USA) using one-way ANOVA for each variable (group, concentration, and time) followed by post hoc Tukey tests. Three-way ANOVA was also used to determine which of the three variables affected cell vitality. Statistical significance was set at *P* < 0.05.

## Results

###  FESEM findings

**Figure 1:**
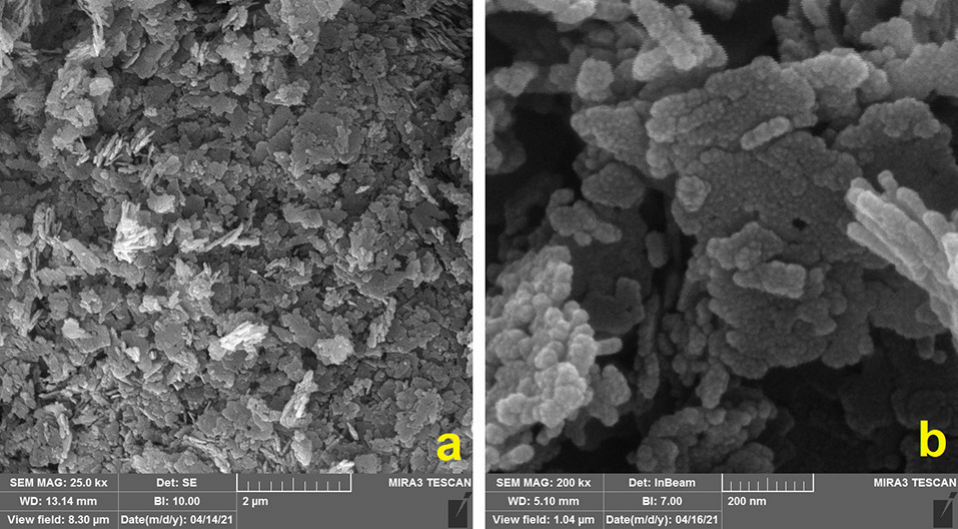
Representative FESEM (field emission scanning electron microscopy) images of zinc oxide nanoparticles at × 25 000 (a) and × 200 000 (b) magnification

**Figure 2:**
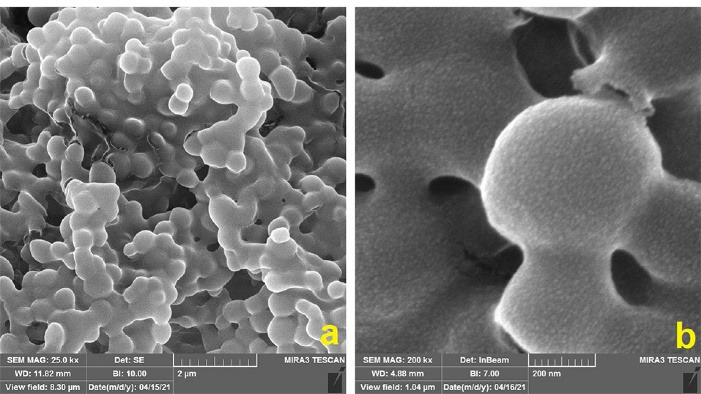
Representative FESEM (field emission scanning electron microscopy) images of polylactic-co-glycolic acid and zinc oxide (PLGA-ZnO) nanocomposite at × 25,000 (a) and × 200,000 (b) magnification

 The FESEM revealed morphological characteristics of the ZnO nanoparticles and the PLGA-ZnO nanocomposite. Figure 1 shows that ZnO nanoparticles are composed of spherical particles with an average diameter of 15 nm. The nanoparticles are generally uniform; however, some agglomeration is noted among the particles. The surface structure of the PLGA-ZnO nanocomposite prepared using the solvent/non-solvent method can be seen in Figure 2. The particles demonstrate uniform spherical shapes with diameters of about 200 nm and smooth surfaces.

###  XRD findings

**Figure 3:**
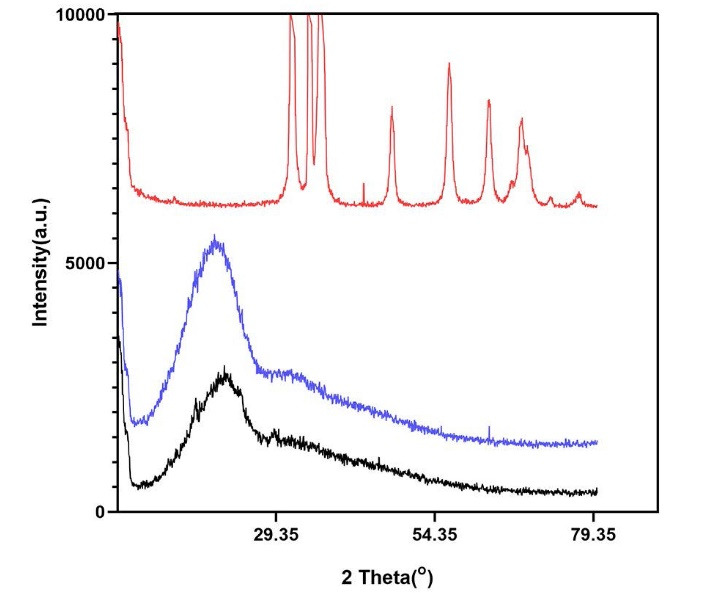
XRD (x-ray diffraction) patterns of ZnO (zinc oxide) in red, PLGA (polylactic-co-glycolic acid) in black, and PLGA-ZnO nanocomposite in blue

 XRD was used to determine the crystalline or amorphous structure of the synthesized nanomaterials. Figure 3 illustrates the XRD patterns of ZnO nanoparticles, PLGA, and PLGA-ZnO nanocomposite. ZnO shows characteristic x-ray diffraction peaks that correspond to the hexagonal wurtzite phase. These peaks reveal the crystalline structure of the ZnO nanoparticles. However, PLGA and PLGA-ZnO nanocomposite only show a broad low-intensity signal at 10‒25°, demonstrating the amorphous structure of these materials. In addition, the intensity of the signal is higher in the nanocomposite than in the PLGA polymer because of the incorporation of metallic nanoparticles in the polymeric matrix.

###  FTIR findings

**Figure 4:**
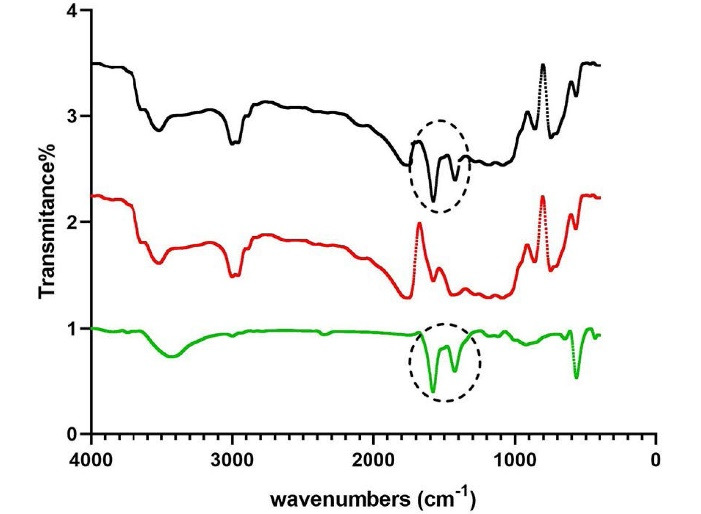
FTIR (Fourier transform infrared) spectrum of ZnO (zinc oxide) in green, PLGA (polylactic-co-glycolic acid) in red, and PLGA-ZnO nanocomposite in black

Figure 4 demonstrates the spectral properties of the nanomaterials. FTIR of ZnO nanoparticles reveals a peak at 463 cm^-1^, related to stretching and bending vibrations of Zn-O bonds, indicating the amount of crystallinity in the synthesized sample. Another peak is noted at 1435 cm^-1^ associated with the stretching vibrations of C = O bonds. FTIR of PLGA shows peaks at 3400‒3500 cm^-1^ (stretching bond of O-H bond), 1700‒1800 cm^-1^ (stretching vibration of C = O bonds), 1100 cm^-1^ (stretching vibration of C-O bonds), and 725 cm^-1^ (bending vibration of C-H bond of the aromatic ring). The FTIR spectrum of PLGA-ZnO nanocomposite is very similar to PLGA; however, it depicts the C = O bond vibration peak at 1435 cm^-1^ similar to the one observed in the ZnO, confirming the presence of ZnO nanoparticles in the polymeric matrix.

###  TGA findings

**Figure 5:**
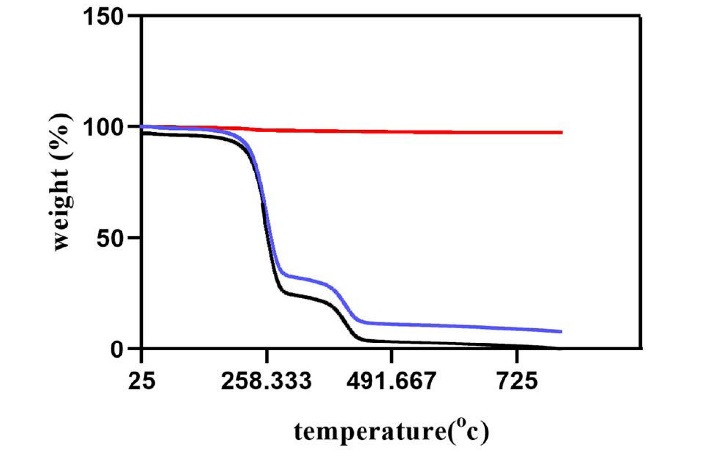
TGA (thermogravimetric analysis) curves of ZnO (zinc oxide) in red, PLGA (polylactic-co-glycolic acid) in black, and PLGA-ZnO nanocomposite in blue

Figure 5 shows the thermal gravimetric analysis for ZnO, PLGA, and the nanocomposite. As you can see, ZnO does not show any weight loss as the temperature increases to 800 °C and is relatively stable even at high temperatures, but PLGA does not behave similarly. The polymer starts decomposing when the temperature reaches 110 °C and loses up to 75% of its total weight at 270 °C. At higher temperatures close to 800 °C, the polymer is completely disintegrated. PLGA-ZnO shows a similar decomposition pattern to PLGA, but the weight loss happens at higher temperatures. The analysis shows that ZnO nanoparticles slightly increase the thermal stability of the PLGA matrix. The nanocomposite starts its degradation at 170 °C, and the weight loss at 270 °C is only 60%. When the temperature reaches 400 °C, 80 wt% loss has occurred in the nanocomposite, but the material is not entirely disintegrated even at temperatures close to 800 °C.

###  MTT findings

Table 1 shows the percentages of viable HGF cells after exposure to different concentrations of ZnO nanoparticles for 24 and 48 hours. The ANOVA analysis revealed that the cell viability was not statistically different from the control group in 24 hours when concentrations of 1 and 20 µg/mL of ZnO were used (*P*= 0.074 and *P*= 0.20, respectively). However, when the concentrations of the metallic nanoparticles were 40 µg/mL or higher, the cytotoxicity increased significantly (*P*= 0.01). Cell viability decreased significantly at all concentrations (1‒100 µg/mL) after 48 hours of exposure to ZnO nanoparticles (*P*= 0.003). The percentage of viable cells after exposure to PLGA polymer is presented in Table 2. The different tested concentrations of PLGA (0.2-20 µg/mL) did not show significant cytotoxicity relative to the control group after 24 hours (*P*= 0.06). This was also true for all PLGA concentrations up to 16 µg/mL after 48 hours of exposure (*P*> 0.05), but PLGA with a concentration of 20 µg/mL showed significant cytotoxicity against HGF cells after 48 hours (*P*= 0.004). However, more than 96% of cells remained vital. Table 3 shows the percentage of cell viability against PLGA-ZnO nanocomposite after 24 and 48 hours. The ANOVA analysis revealed that at 24 hours, cell viability was similar to the control group for all concentrations up to 80 µg/mL (*P*> 0.05). However, when 100 µg/mL of ZnO was incorporated into the PLGA matrix, the cytotoxicity was statistically higher than the control group (*P* < 0.001). Toxicity increased at 48 hours and was noteworthy for concentrations of ≥ 60 µg/mL (*P* < 0.001). By adjusting the effect of each other, the simultaneous effect of interventions (PLGA, ZnO, and PLGA-ZnO), groups (different concentrations), and time (24 and 48 hours) was significant, according to the results of the three-way ANOVA analysis (*P* < 0.001).

**Table 1:** Viability of human gingival fibroblasts treated with zinc oxide nanoparticles

**Zinc oxide concentration**	**Time (h)**	**Cell viability (%)** **Mean±SD**
Control	24	99.75 ± 0.50
	48	99.75 ± 0.50
1 µg/mL	24	98.42 ± 1.54
	48	95.22 ± 3.01
20 µg/mL	24	97.31 ± 1.82
	48	94.91 ± 3.20
40 µg/mL	24	95.90 ± 2.00
	48	93.38 ± 1.86
60 µg/mL	24	90.69 ± 2.51
	48	90.48 ± 2.13
80 µg/mL	24	86.84 ± 1.65
	48	83.41 ± 1.46
100 µg/mL	24	78.84 ± 2.42
	48	71.31 ± 1.83

**Table 2:** Viability of human gingival fibroblasts treated with Polylactic-co-glycolic acid (PLGA)

**PLGA concentration**	**Time (h)**	**Cell viability (%)** **Mean±SD**
Control	24	99.75 ± 0.50
	48	99.75 ± 0.50
0.2 µg/mL	24	98.73 ± 1.48
	48	98.52 ± 1.54
4 µg/mL	24	97.77 ± 1.74
	48	98.16 ± 2.03
8 µg/mL	24	97.20 ± 1.23
	48	97.90 ± 1.69
12 µg/mL	24	98.47 ± 0.55
	48	97.87 ± 1.23
16 µg/mL	24	98.75 ± 0.85
	48	98.47 ± 1.33
20 µg/mL	24	96.79 ± 2.80
	48	96.31 ± 1.17

**Table 3:** Viability of human gingival fibroblasts treated with Polylactic-co-glycolic acid and Zinc oxide (PLGA-ZnO) nanocomposite

**PLGA-ZnO concentration**	**Time (h)**	**Cell viability (%)** **Mean±SD**
Control	24	99.75 ± 0.50
	48	99.75 ± 0.50
0.2 µg/mL PLGA + 1 µg/mL ZnO	24	96.71 ± 3.00
	48	98.14 ± 2.33
4 µg/mL PLGA + 20 µg/mL Zn	24	97.11 ± 1.51
	48	98.19 ± 2.04
8 µg/mL PLGA + 40 µg/mL ZnO	24	96.37 ± 2.66
	48	97.66 ± 1.78
12 µg/mL PLGA + 60 µg/mL ZnO	24	98.03 ± 1.03
	48	93.45 ± 2.49
16 µg/mL PLGA + 80 µg/mL ZnO	24	96.51 ± 2.01
	48	92.35 ± 2.21
20 µg/mL PLGA + 100 µg/mL ZnO	24	94.25 ± 1.03
	48	89.67 ± 2.71

## Discussion

 In this study, we evaluated the cytotoxicity of different concentrations of PLGA-ZnO nanocomposite on HGF in 24 and 48 hours. ZnO nanoparticles have been extensively investigated for their antibacterial qualities. This property could be beneficial for application within wound dressings.^19-21^ These nanoparticles are relatively safe when used at low concentrations but can result in significant cell death at high doses. Only 71% of the HGF cells remained viable after 48 hours of exposure to 100 µg/mL of ZnO, and the remaining cells lost their vitality. This can be a crucial concern when considering the biocompatibility of these metallic nanoparticles.

 One of the primary mechanisms involved in the cytotoxicity of ZnO nanoparticles is the release of Zn^2+^ ions. Since the surface potential of most cells is negative, the positively charged ions are attracted toward the cell membranes. They can penetrate the cell, damage the mitochondria, and disrupt homeostasis, leading to cell death.^22^ As the results indicated, incorporating ZnO nanoparticles into the PLGA matrix reduces their toxicity against HGF. Furthermore, integrating these nanoparticles into the polymeric matrix decreases the number of free zinc ions released, which can explain the lower cytotoxicity of PLGA-ZnO nanocomposite compared to ZnO nanoparticles.

 The cytotoxicity of ZnO nanoparticles is time- and dose-dependent. Şeker et al^10^ evaluated the cytotoxicity of different concentrations of ZnO on mouse dermal and human PDL fibroblasts in 6 to 48 hours. They concluded that using up to 50 µg/mL of ZnO nanoparticles is safe and does not induce significant cytotoxicity. However, higher doses should be used with caution. In contrast, Vergara-Llanos et al^23^ reported biocompatibility at 78‒100 µg/mL concentrations of ZnO nanoparticles against HGF after 24 hours of exposure. However, in our study, significant cell death was observed when ZnO concentrations exceeded 40 µg/mL. Stankovic et al. 18 studied the safety of PLGA-ZnO nanocomposite on human hepatoma HepG2 cells in 24 hours, concluding that this material is safe at concentrations as high as 0.01% w/v, equivalent to 100 µg/mL. The cytotoxicity of PLGA-ZnO nanocomposite was investigated in another study by Burmistrov et al^9^ on human neuroblastoma SH-SY5Y cells treated for 72 hours. They observed that PLGA alone did not induce significant cell damage but incorporating 0.1% concentration of ZnO nanoparticles into the PLGA matrix reduced the number of viable cells.

 Various methods have been proposed for manufacturing the PLGA-ZnO nanocomposite.^9,24,25^ The hydrothermal process using the solvent/non-solvent method is a straightforward and environmentally friendly process that yields more control over the morphology of the nanoparticles since it leads to the formation of uniform spherical particles with a mean diameter of 30‒40 nm.^18,26,27^ XRD, TGA, and FTIR analyses are standard protocols that, as explained in the results, demonstrate the effective incorporation of the ZnO nanoparticles into the PLGA matrix. Since ZnO nanoparticles can increase the stability of the PLGA polymer, as indicated by TGA analysis, this combination can also be beneficial for the polymeric matrix. Furthermore, by adding ZnO nanoparticles, the disordering and loosening of the chains in PLGA polymer decrease, and the material becomes more stable against decomposition, leading to sustained release of the metallic nanoparticles, which can also explain the reason for zinc oxide toxicity reduction when added to the polymeric matrix.^9^ Therefore, the combination of PLGA with ZnO can be an effective strategy for reducing the toxicity of ZnO nanoparticles against gingival fibroblasts to safely use this antibacterial agent in wound dressings after periodontal surgery without harming the gingival tissue.

 There were some limitations to this investigation. First, the duration of exposure of HGF cells to the nanomaterials was limited to 24 and 48 hours. Long-term cell exposure to these materials should be investigated to establish their biocompatibility. This study only tested the biocompatibility of PLGA-ZnO nanocomposite on HGF cells; however, many different cells are involved in wound healing. The nanomaterial has to be safe against all the cells engaged in the healing process to prevent interfering with it. Different doses of these nanomaterials must be evaluated to determine the optimum dose that would be safe for human cells and efficient against oral pathogenic bacteria.

## Conclusion

 Within the limitations of this study, we can conclude that zinc oxide nanoparticles are toxic against human gingival fibroblasts at concentrations ≥ 40 µg/mL. The toxicity can be reduced by incorporating these metallic nanoparticles in the PLGA matrix, so the combination of PLGA with ZnO could be a better option for adding to wound dressings after periodontal surgery; however, more in vitro and in vivo studies are required before this nanocomposite can be used in daily practice.

## Acknowledgments

 The authors express their gratitude to Mahamax Laboratory (Tehran, Iran) for their assistance in conducting the laboratory procedures.

## Availability of Data

 The datasets used and/or analyzed during the current study are available from the corresponding author upon reasonable request.

## Competing Interests

 The authors declare that they have no competing interests related to authorship and/or the publication of this work.

## Ethical Approval

 This research was approved by the Research Ethics Committee of Qazvin University of Medical Sciences (IR.QUMS.REC.1400.326).

## Funding

 The study was self-funded by the corresponding author.
